# Potassium urinary excretion and dietary intake: a cross-sectional analysis in 8–10 year-old children

**DOI:** 10.1186/s12887-015-0374-z

**Published:** 2015-05-17

**Authors:** Ana Catarina Oliveira, Patrícia Padrão, André Moreira, Mariana Pinto, Mafalda Neto, Tânia Santos, Joana Madureira, Eduardo de Oliveira Fernandes, Pedro Graça, João Breda, Pedro Moreira

**Affiliations:** 1 Faculty of Nutrition and Food Sciences, University of Porto, R. Dr. Roberto Frias, Porto, 4200-465 Portugal; 2 Institute of Public Health – University of Porto (ISPUP), Porto, Portugal; 3 Department of Immunology, Faculty of Medicine, University of Porto, Porto, Portugal; 4 Department of Immunoallergology, Hospital of São João, Rua Prof. Hernâni Monteiro, Porto, 4200-319 Portugal; 5 Faculty of Sciences, University of Porto, Porto, Portugal; 6 Institute of Mechanical Engineering, Faculty of Engineering, University of Porto, Porto, Portugal; 7 Directorate General for Health (Direcção Geral de Saúde), Lisbon, Portugal; 8 Division of Noncommunicable Diseases and Life-course, WHO Regional Office for Europe, UN City, Copenhagen, Denmark; 9 Research Centre on Physical Activity and Health, University of Porto, Rua Dr. Plácido Costa, 91, Porto, 4200-450 Portugal

**Keywords:** Potassium, Sodium, Children, 24-h urinary excretion, 24-h dietary recall questionnaire, Fruit and vegetable

## Abstract

**Background:**

Data from studies assessing the intake of potassium, and the concomitant sodium-to-potassium ratio are limited. The aim of this study was to evaluate potassium and sodium-to-potassium ratio intake in 8–10 year-old children.

**Methods:**

A cross-sectional survey was carried out from January to June 2014 and data from 163 children (81 boys) were included. Potassium intake was estimated by 24-h urine collection and coefficient of creatinine was used to validate completeness of urine collections. Urinary sodium and sodium-to-potassium ratio were also analysed. A 24-h dietary recall was used to provide information on dietary sources of potassium. Height and weight were measured according to international standards.

**Results:**

The mean urinary potassium excretion was 1701 ± 594 mg/day in boys, and 1682 ± 541 mg/day in girls (*p* = 0.835); 8.0 % of children met the WHO recommendations for potassium intake. The mean sodium excretion was 2935 ± 1075 mg/day in boys and 2381 ± 1045 mg/day in girls (*p* <0.001) and urinary sodium-to-potassium ratio was 3.2 ± 1.4 in boys, and 2.5 ± 1.1 in girls (*p* = 0.002). The mean fruit and vegetable intake was 353.1 ± 232.5 g/day in boys, and 290.8 ± 213.1 g/day in girls (*p* = 0.101).

**Conclusions:**

This study reported a low compliance of potassium intake recommendations in 8–10 year-old children. Health promotion interventions are needed in order to broaden public awareness of potassium inadequacy and to increase potassium intake.

## Background

According to the World Health Organization (WHO), non-communicable diseases (NCDs) are the main contributor to mortality and morbidity globally, killing more people each year than all other causes combined [1]. Because of the increasing prevalence of NCDs, dietary guidelines designed to promote public health have been instituted worldwide as an important part of national food and nutrition policies [2]. Interventions that are well understood, cost effective, and feasible could counteract NCDs [3], since the most health-promoting diets do not require extraordinary effort, cost, or complexity and provide the recommended daily allowances of macro and micronutrients [4]. Potassium (K^+^) is one of the key nutrients to prevent NCDs, and to preserve total body fluid volume, acid and electrolyte balance, and normal cell function [1, 3]. WHO suggests to adjust downward the recommended potassium intake of at least 3510 mg/day, based on the energy requirements of children relative to those of adults [1, 3]. Food rich in K^+^, such as fruits and vegetables [1], are recognized as key contributors to a healthy diet and are in line with food based dietary guidelines [5–7]. In addition, processed foods are often lacking K^+^ [1, 3].

Most NCDs are chronic diseases and have slow progression [8]. Although these diseases disproportionately affect adults, they are now being detected more frequently in paediatric populations, as well as their risk factors [3]. Furthermore, diet in childhood can be a significant determinant of adult dietary habits even after several decades [2, 9].

Major NCDs include cardiovascular diseases, cancers, chronic respiratory diseases and diabetes [8]. High blood pressure (BP) or hypertension [10, 11] is a major risk for cardiovascular diseases, especially coronary heart disease, stroke and heart failure [3], and are of increasing concern in children and adolescents [12]. Primary hypertension probably has its onset in the first decades of life [13, 14].

Diet is believed to play an important role in hypertension [15, 16], especially one high in sodium (Na^+^) and low in K^+^ content [17]. The part played by dietary factors has been thoroughly studied [17]. Na^+^ intake has been the main topic, but most studies have been concerned with adults [13]. The association between high intake of Na^+^ and increase of BP [12, 18], as well as hypertension is well established [19]. Intake of Na^+^ is also associated with significantly increased risk of stroke and other cardiovascular diseases [20–22]. A diet that includes modest salt restriction serves as a strategy to prevent or control hypertension and decrease cardiovascular morbidity and mortality [12, 18, 20, 21, 23]. However, this issue is not consensual as some authors argue that blood pressure effect of sodium restriction on blood pressure can no longer be accepted as a surrogate for health outcomes [24] and both low sodium intakes and high sodium intakes may be reported has being associated with increased cardiovascular risk [25] and mortality [26].

The potential role of the intake of K^+^ on BP has received much less attention than sodium [27] although much evidence shows that increasing K^+^ intake has beneficial effects on human health [28]. It was reported that K^+^ intake was associated with a lower risk of stroke [3, 29, 30] and hypertension [4, 19, 31], since it lowers the BP [3, 27]. However, the evidence on the potential beneficial effect of increased K^+^ on cardiovascular disease is not entirely consistent [3], and confounding effects may arise from the interaction among various dietary electrolytes, in particular, the Na^+^/K^+^ ratio and other nutrients in K^+^ rich foods [13]. As Na^+^ consumption rises, increased consumption of K^+^ may be even more beneficial because, in addition to other benefits, it can mitigate the negative effects of elevated Na^+^ consumption on BP [32]. Furthermore, when people have an increased intake of K^+^, high intake of Na^+^ may not be associated with higher BP [33]. Also, regarding dietary intake, high consumption of fruit and vegetables, important sources of potassium, is associated with a lower risk of all-cause mortality, particularly cardiovascular mortality [34] and stroke [35].

However, data from studies conducted in children are limited, particularly those which assess both the intake of Na^+^ and K^+^, and the concomitant ratio in health outcomes [1]. The available evidence suggests that increased K^+^ intake might reduce BP or at least prevent a deleterious rise in BP with age [36]. However, more studies are needed to verify the effects of K^+^ intake on BP and study its potential adverse effects in children [1].

The best method to estimate K^+^ intake is 24-h K^+^ excretion in urine, as the use of dietary surveys and food composition databases for estimating K^+^ intake may introduce either an over or underestimation of the actual intake [5, 37–39]. Increased urinary K^+^ is reported to be associated with a lower body mass index (BMI), diastolic BP and heart rate, as well as lower stroke risk [30]. A better diet quality may also be associated with lower Na^+^ excretion and higher K^+^ excretion in six-year-old children [5].

Since K^+^ intake can be a predictor of overall dietary quality and can lead to beneficial effects on human health, we aimed to evaluate and provide information on consumption levels of K^+^ by 8–10 year-old school children, measured by 24-h urinary excretion, and assess dietary sources of potassium.

## Methods

### Study population

The cross-sectional survey, which was conducted between January and June 2014, included elementary school children (8–10 year olds), in Portugal.

To accomplish this project parents and caretakers of 488 children, attending the 3rd and 4th grade, were contacted in person. Details about the study were explained, including why the research was being conducted, what the study involved, the methods and procedures used, and the contact details for any necessary support. Participants were also informed that participation was voluntary and that they were able to withdraw at any time. All students and their parents received written information on the project. Of the 202 (41.4 %) children who agreed to participate, all collected a 24-h urine sample, but 33 (16.3 %) were excluded for incomplete 24-h urine collection according to the coefficient of creatinine (described in detail below). All the children (n = 202) responded to the 24-h dietary recall questionnaire, although six children were considered under-reporters (four girls) according to Goldberg cut-offs. Given that from these six children, five have already been excluded for not exhibiting valid urine samples, only one child was further excluded, remaining a sample of 168 participants. From these, five children (one with seven years old and four with 11) were also further excluded to narrow the age-range of the subjects, remaining a final sample of 163 participants (82 girls).

Prior data collection, parents provided written informed consent, according to the ethical standards laid down in the Declaration of Helsinki, and children gave oral assent. All schools, where the study was carried out, and the Ethical Committee of the University of Porto approved the protocol of study.

### General data collection

Data collection was done through structured interviews, anthropometric measures, questionnaires, and collection of 24-h urine sample and 24-h dietary recall by trained researchers.

### Socioeconomic variables

All parents and caretakers underwent a structured questionnaire to collect information on sociodemographic characteristics, namely age and sex, and parental education level (recorded in 3 categories: 0–9 years; 10–12 years; >12 years).

### Anthropometric measures

The collection of anthropometric data followed the standardized procedures [40]. Body weight measurement was obtained using an electronic scale (TANITA® TBF-300A, capacity 200 kg, accuracy 100 g) and the height was obtained using a stadiometer (capacity 200 cm, accuracy 1 mm) with the head in the Frankfort plane [41]. Participants wore lightweight clothing and no shoes. Body mass index (BMI) was computed as mass, (kg)/height^2^, (m). After calculating the BMI, it was plotted on the WHO BMI-for-age growth charts and obtained a percentile ranking, classifying children as: underweight (less than the 3rd percentile), normal weight (3rd to less than the 85th percentile), overweight (85th to less than the 97th percentile) or obese (equal to or greater than the 97th percentile) [42].

### Physical activity

Physical activity was measured by a questionnaire sent to each parent or caretaker. This questionnaire included: the time spent watching TV/video during most days of the week (<2 h/day; and ≥2 h/day); sleeping duration (≤8 h/day; 9 h/day; and ≥10 h/day); and practice of sports activities besides the physical education classes at school (<2 times/week; 2–3 times/week; >4 times/week) [43].

### Urine collection

Parents and caretakers were given both verbal and written instructions in assisting children to collect a 24-h urine sample and received a standard sterilized urine collection bottle. On the first morning of the urine collection, instructions were given to discard the first specimen, and from then on to collect all specimens for up to 24 h, including the first specimen of the following day. The samples were analysed by certified laboratories for 24-h creatinine (mg/day), 24-h urine volume (mL), 24-h sodium (mEq/day), and 24-h potassium (mEq/day). Urinary Na^+^ and urinary K^+^ were converted to mg/day (23 mgNa^+^ = 1 mmol Na^+^ or 1 mEq Na^+^; 39 mgK^+^ = 1 mmol K^+^ or 1 mEq K^+^) [44].

The 24-h urine collections were assessed for completeness using creatinine excretion in relation to weight (i.e. creatinine coefficient), calculated by the following formula:

$Creatinine\kern0.5em  coefficient = \frac{Creatinine\kern0.5em \left( mg/ day\right)}{Body\  weight\ (kg)}$. Creatinine coefficients above of 0.1 mmol · kg^−1^ · day^−1^ were classified as indicating an acceptable 24-h urine collection [45].

### Dietary assessment

When participants delivered the 24-h urine collection, a 24-h dietary recall questionnaire [46] was filled, taking into account the *Manual de Quantificação dos Alimentos* [47]. Participants were questioned accurately about their food and drinks consumption, even reporting cooking methods, brands and consuming time and place. The software *Food Processor*®, (ESHA Research, USA) was used to convert food into nutrients.

To identify under-reporters, Goldberg cut-offs were used as direct comparison of energy intake (EI) to energy expenditure [48]. The Goldberg cut-off values were applied to exclude under-reporters, based on PAL (Physical Activity Level) and compared with the ratio of EI to BMR (Basal Metabolic Rate). BMR was calculated using the Schofield equations for children based on age, gender, height and weight [49].

While the principles of the Goldberg et al. [50] cut-offs still hold when assessing the EI of children and adolescents, appropriate age- and gender-specific cut-offs should always be applied in a pediatric population [51]. Therefore, according to the formulas proposed by Goldberg et al. [50], we calculated individual “CUTOFF 2” values using coefficients of variation for BMR of 8.5 %, coefficients of variation for EI (23 %) given by Nelson et al. [52], and published levels of light physical activity (1.55 for boys and 1.50 for girls for this age group) given by Torun et al. [53]. We used these estimated limits specific for age and sex instead of the single “CUT-OFF 2” for adults as proposed by Goldberg et al. Thus, records with EI:BMR ratios up to 0.95 for boys and 0.92 for girls were considered not plausible records. This result is in agreement with Sichert-Hellert et al. [54], although differences may occur due to the number of days of dietary assessment (one versus three days).

Finally, diet recall data was analysed and grouped in order to assess potassium rich foods consumption: milk and whey-based milk products; pulses; vegetables; fruit; and fruit and vegetables [55]. We also considered high and low intake of these food groups based on, respectively, intakes at or above the median, and below the median.

### Statistical analysis

Statistical analysis was conducted using SPSS Statistical Package® 21.0 (IBM Corporation, 2012).

Continuous variables were presented as mean and standard deviation, and percentiles, and categorical variables were summarized as counts and percentages.

Kolmogorov – Smirnov test was performed to test variables for normality. Independent samples *t*-test (parametric variables) and non-parametric test (Mann–Whitney U) were used to identify sex differences for sodium and potassium excretion. Categorical variables were tested using the Chi-square test. A univariate General Linear Model (GLM) was performed to identify sex differences for nutritional and dietary intake and we used the energy intake as a covariate, except for variables expressed as % of total energy intake (TEI) or g/1000 kcal.

We also used independent sample *t*-test, ANOVA, and GLM to investigate the associations between potassium excretion and participants’ characteristics (BMI categories, sports activities, television/video viewing and father’s and mother’s education), and food groups consumption, using energy intake as a covariate in GLM.

A *p*-value <0.05 was considered to indicate statistical significance.

## Results

Table 1 shows the characteristics of participants. Half of the participants were eight years old, and nearly one third was nine years old.

**Table 1:** Characteristics of participants

		Boys n (%)	Girls n (%)	*p*
Age (years)	8	40 (49.4 %)	46 (56.1 %)	
	9	30 (37.0 %)	24 (29.3 %)	
	10	11 (14.8 %)	12 (14.6 %)	0.570
School grade	3rd grade	47 (68.1 %)	50 (72.5 %)	
	4th grade	22 (31.9 %)	19 (27.5 %)	0.355
BMI categories	Underweight	2 (2.5 %)	4 (4.9 %)	
	Normal weight	46 (56.8 %)	46 (56.1 %)	
	Overweight	10 (12.3 %)	18 (22.0 %)	
	Obese	23 (28.4 %)	14 (17.1 %)	0.162
Sports activities (times/week)	<2/week	31 (45.6 %)	44 (61.1 %)	
	2–3/week	29 (42.6 %)	24 (33.3 %)	
	> 4/week	8 (11.8 %)	4 (5.6 %)	0.047*
Sleeping (hours/day)	≤8 h/day	23 (33.3 %)	16 (22.9 %)	
	9 h/day	26 (37.7 %)	29 (41.4 %)	
	≥10 h/day	20 (29.0 %)	25 (35.7 %)	0.374
Television/video viewing	<2 h/day	62 (91.2 %)	60 (93.8 %)	
	≥2 h/day	6 (8.6 %)	4 (6.3 %)	0.411
Father’s education	0–9 years	34 (57.6 %)	28 (45.2 %)	
	10–12 years	12 (20.3 %)	22 (35.5 %)	
	>12 years	13 (22.0 %)	12 (19.4 %)	0.175
Mother’s education	0–9 years	35 (53.9 %)	29 (45.3 %)	
	10–12 years	14 (21.5 %)	16 (25.0 %)	
	>12 years	16 (24.6 %)	19 (29.7 %)	0.623

*Calculated for categories <2/week and ≥2/week

Most children were within the normal range of BMI for age and sex (56.4 %), and no significant difference across BMI categories between sexes was found (*p* = 0.162).

Girls were significantly less involved in physical activities than boys (61.1 % of girls reported exercise <2 times/week versus 45.6 % of boys, *p* = 0.047). Sleep duration for less than 8 h/day was reported by 33.3 % of boys, and 22.9 % of girls. The proportions of children who spent two or more hours watching TV/video during most days of the week were 8.6 % in boys and 6.3 % in girls.

Nearly half of parents had attended school until nine years and only a small percentage completed 10–12 years or a longer school education.

The average K^+^ excretion was similar in both sexes (1701 ± 594 mg/day for boys and 1682 ± 541 mg/day for girls, *p* = 0.835). The average Na^+^ urinary excretion was significantly higher in boys (2935 ± 1075 mg/day vs. 2381 ± 1045 mg/day in girls, *p* <0.001), as well as the mean urinary Na^+^/K^+^ ratio (3.2 ± 1.4 vs. 2.5 ± 1.1, in girls, *p* = 0.002). Table 2 summarizes descriptive data on Na^+^ and K^+^ excretion.

**Table 2:** Sodium excretion, potassium excretion and sodium-to-potassium ratio by sex

	Sodium (mg)		Potassium (mg)		Sodium-to-potassium ratio	
	Boys	Girls	Boys	Girls	Boys	Girls
Percentiles						
5	1495	1233	747	909	1.50	1.21
25	2093	1737	1297	1287	2.18	1.82
50	2737	2104	1658	1560	2.73	2.33
75	3749	2760	1979	2077	3.75	2.91
95	4996	4685	2982	2796	6.55	4.65
Mean	2935	2381	1701	1682	3.16	2.52
St. Deviation	1075	1045	594	541	1.42	1.05
Minimum	1150	1012	429	780	1.13	1.01
Maximum	6141	6647	3627	3003	8.18	6.94
*p*	<0.001		0.835		0.002	

The overall mean energy intake was 2262 ± 555 kcal for boys and 2117 ± 547 kcal for girls (*p* = 0.098). Table 3 shows macronutrient intake and potassium rich foods consumption by sex, adjusted for energy intake.

**Table 3:** Nutritional and dietary intake by sex

	Boys (n = 81)		Girls (n = 82)		*p*
	Mean	SD	Mean	SD	
Energy (kcal)	2261.7	554.6	2116.9	546.5	0.098
Protein (%TEI)	16.7	3.5	18.1	4.5	0.050
Total fat (%TEI)	28.7	6.6	28.7	6.2	0.569
SFA (%TEI)	9.1	3.1	8.9	3.1	0.778
MUFA (%TEI)	10.4	3.1	9.9	3.2	0.555
PUFA (%TEI)	3.6	1.6	3.6	1.6	0.892
Total CHO (%TEI)	51.6	7.3	50.0	7.9	0.092
Sugarsª (%TEI)	19.4	6.4	18.7	7.4	0.427
Fibre (g/day)	16.1	5.8	16.8	8.4	0.094*
Fibre (g/1000 kcal)	7.2	2.4	7.9	3.1	0.160
Cholesterol (mg)	269.3	121.1	272.9	153.4	0.369*
Milk and whey-based milk products (g/day)	484.7	283.8	515.8	267.8	0.125*
Pulses (g/day)	24.0	99.1	22.5	55.0	0.941*
Vegetables (g/day)	153.0	138.3	114.4	120.7	0.078*
Fruit (g/day)	219.7	170.7	192.0	146.2	0.386*
Fruit and vegetables (g/day)	353.1	232.5	290.8	213.1	0.101*

*CHO* carbohydrates, *SFA* saturated fatty acids, *MUFA* monounsaturated fatty acids, *PUFA* polyunsaturated fatty acids, *TEI* total energy intake

ªSugars refer to all monosaccharides and disaccharides added to foods by the manufacturer, cook or consumer, plus sugar naturally present in honey, syrups, and fruit juices

*Adjusted for energy intake

Table 4 shows the relation between urinary potassium excretion and descriptive variables. After considering the mean urinary potassium excretion according to BMI categories, exercise, parents’ education, and time spent watching television/video viewing no significant differences were found for potassium excretion. Moreover, no significant differences were found for potassium excretion according to potassium rich food group’s consumption.

**Table 4:** Relation between urinary potassium excretion and descriptive variables

		Potassium excretion (mg/day)			
		Boys (n = 81)		Girls (n = 82)	
		Mean	SD	Mean	SD
BMI categories	Underweight	1618.5	82.7	1540.5	622.0
	Normal weight	1601.5	573.9	1644.1	577.1
	Overweight	1924.0	826.9	1670.5	537.5
	Obese	1807.0	534.5	1841.4	437.0
p		0.241		0.582	
p*		0.248		0.373	
Sports activities (times/week)	<2/week	1737.4	477.7	1663.7	520.5
	2–3/week	1664.0	668.8	1666.8	652.4
	>4/week	1359.4	652.8	1560.0	315.2
p		0.281		0.886	
p*		0.200		0.259	
Sleeping (hours/day)	≤8 h/day	1665.1	485.0	1642.9	386,0
	9 h/day	1629.5	764.8	1710.4	682,8
	≥10 h/day	1696.5	465.5	1648.9	497,9
p		0.805		0.765	
p*		0.143		0.091	
Television/	<2 h/day	1642.6	537.3	1664.7	557.3
	≥2 h/day	1859.0	969.4	1911.0	565.2
p		0.440		0.261	
p*		0.638		0.104	
Father’s education	0–9 years	1673.6	407.3	1571.1	481.9
	10–12 years	1960.6	616.2	1654.7	549.8
	>12 years	1578.0	875.9	1547.0	509.1
p		0.271		0.800	
p*		0.144		0.376	
Mother’s education	0–9 years	1655.8	426.4	1540.5	451.3
	10–12 years	1818.0	688.6	1881.8	499.1
	>12 years	1716.0	774.1	1521.0	557.5
p		0.712		0.072	
p*		0.612		0.784	
Milk and whey-based milk products (g/day)	Low^a)^	1684.6	14.8	1678.2	16.3
	High	1693.8	21.8	1689.8	19.3
p		0.231		0.036	
p*		0.373		0.060	
Pulses (g/day)	Low ^a)^	1689.3	18.8	1681.1	16.8
	High	1688.1	20.3	1691.0	21.4
p		0.507		0.767	
p*		0.476		0.944	
Vegetables (g/day)	Low ^a)^	1686.8	23.7	1682.6	21.7
	High	1690.9	14.0	1685.9	14.0
p		0.587		0.301	
p*		0.622		0.366	
Fruit (g/day)	Low ^a)^	1687.6	15.6	1683.1	19.2
	High	1690.7	22.5	1685.1	18.3
p		0.955		0.178	
p*		0.963		0.195	
Fruit and vegetables (g/day)	Low ^a)^	1689.0	23.5	1683.4	21.6
	High	1689.0	15.2	1684.9	14.9
p		0.972		0.260	
p*		0.890		0.299	

*Adjusted for energy intake

^a)^ Low and high refers to intakes below the median or at or above the median, respectively

## Discussion

Average daily K^+^ intake in children was much lower than the recommended level in both sexes, and only 8.0 % of the children had a K^+^ excretion corresponding to an intake equal or higher than the recommended by WHO, considering energy requirements by age and sex, [1] which suggests a low diet quality. Accordingly, the mean consumption of fruit and vegetables was below the recommended intake level [56]. When comparing the urinary excretion of K^+^ with the corresponding level of the Dietary Reference Intake - Adequate Intake (AI) which is 4.5 g K^+^/day [57], we did not found any children above this level of intake.

The mean K^+^ excretion in the present study was 1681 mg/day. According to our best knowledge, only two studies [5, 14] addressed the K^+^ urinary excretion in school children, although none had studied children from the age range of the present study. Kristbjornsdottir et al. examined six year-old children and reported excretion of about 1210 mg/day [5] while Allison et al. who studied 3–5 years-old children showed a K^+^ excretion of 1000 mg/day [14]. Nevertheless, it is often challenging to compare values from studies on children, mainly due to the different nutritional requirements, [5] particularly when considering K^+^ recommendations depending on energy needs [5].

The mean Na^+^ excretion in this study was 2657 mg/day, and only 7.4 % of the participants had complied with the recommended by WHO, considering age, sex, and energy requirements. On the other hand, only 9.8 % of the children did not exceed the AI of Na^+^ [57]. Moreover 54.0 % of the children exceeded the Tolerable Upper Intake Level for sodium [57].

There is also a need to evaluate urinary Na^+^ and the Na^+^/K^+^ ratio considering their interrelated biological effects beyond their absolute levels of intake [7]. In this study, the mean Na^+^/K^+^ ratio was 2.8 ± 1.29. Although WHO do not address optimal Na^+^/K^+^ ratio, it is suggested that if recommendations of both Na^+^ and K^+^ consumption are achieved, the molar ratio of Na^+^/K^+^ would be approximately one to one, which is considered beneficial for health [1, 56]. In this sample, the mean Na^+^/K^+^ ratio was nearly three times higher than the favourable level for health, in line with other authors. A study conducted in southern Italy with subjects aged 2–16 years showed an average Na^+^/K^+^ ratio of 3.79 ± 1.68 [58] while in another survey with French participants aged 2–14 years the ratio was 1.64 [6]. Other studies showed that there is a low K^+^ and high Na^+^ intake in children [6, 59, 60] suggesting a low consumption of potassium rich foods.

In this study a high potassium excretion does not seem a marker of a healthy lifestyle since low levels of physical activity (*p* = 0.28 for boys and *p* = 0.89 for girls), high sedentary time (*p* = 0.44 for boys and *p* = 0.26 for girls) and body overweight status (*p* = 0.24 for boys and *p* = 0.58 for girls) were not significantly associated with urinary potassium excretion. Potassium excretion is not significantly higher in children that consume more fruit and vegetables, both for boys (*p* = 0.992) and girls (*p* = 0.721), as it would be expected. The difficulty in reporting accurately vegetable consumption specially in food preparations in which vegetables are triturated, such as soups (a staple item of the Portuguese diet), may contribute to explain this unexpected observation.

However, when assessing dietary intake, particularly on children and adolescents, measurements are prone to error reports, [2] namely inaccuracies in self-reporting, incorrect or incomplete food composition tables, missing data [37], attenuation from daily variation in nutrient intake [61] and mostly through under-reporting [62, 63] Although the seven days food record method had the highest correlation coefficients when compared with nutritional biomarkers [61], we performed a 24-h dietary recall to estimate major K^+^ rich foods intake, after excluding misreporters for energy. The estimated dietary potassium intake and urinary potassium excretion were in agreement, although their correlation was a weak positive one (*r* = 0.039), which may be explained by the inaccuracy in dietary reporting in children.

In this study, the intake of fibre was lower than the recommended [64], which may reflect the low contribution of K^+^ rich foods such as fruit, vegetables and pulses. The mean contribution of protein to total energy intake for boys and girls was above the recommended, while the mean contribution of carbohydrate to total energy intake for boys and girls was below the recommended range and the mean contribution of fat to total energy intake was within the recommended range for boys and girls [56]. Saturated fatty acids (SFA) was slightly higher than the recommended, while the mean energy intake from polyunsaturated fatty acids (PUFA) was much lower than the recommended [65]. These results are close to other results described for Portuguese children [66, 67].

The high Na^+/^K^+^ ratio found in this study may also reflect the low proportion of children (33.7 %) who achieved the recommendation to eat at least 400 g of fruit and vegetables/day [56] which is in line with other studies [2, 68]. The importance of fruit and vegetables as sources of K^+^ [29, 35] raises the question of the inadequacy of food habits in children from a Mediterranean country, concomitantly with the consumption of too salty foods. However, social desirability and the possibility of these children not knowing some vegetables, fruits or pulses may also have affected the estimation of these items by the 24 h dietary recall used in our study.

The best way to increase K^+^ intake is to consume more fruits and vegetables [28] which could have other beneficial effects, besides increasing K^+^ intake, namely on antioxidants micronutrients and fibre [28, 35].

The results of this study point out the urgent need to develop and implement policies aimed at reducing Na^+^ intake and increasing K^+^ intake at this specific population and underscore the need for population-based interventions, such as consumer education, improvement of product labelling, changes in food production and restaurant offerings [39]. The evaluation of population current dietary Na^+^ and K^+^ intakes is needed to measure the success of future intervention strategies [19].

The main strengths of this study are the use of 24-h urine collection for the assessment of K^+^ and Na^+^ intake, as well as the Na^+^/K^+^ ratio, in accordance with WHO recommendations that the assessment of Na^+^ should be followed by the information of K^+^ [69]. Furthermore, the diet and nutritional intake estimated by the 24 h dietary recall are also presented in this report, providing information on K^+^ food sources, which may facilitate the design of preventive strategies.

There are some limitations as well. The fact that we only had one 24-h urine excretion per individual might be considered a limitation, as more than one collection may be needed for individual assessment of Na^+^ and K^+^ excretion due to day-to-day within-person variability of K^+^ intake. Another limitation is dietary assessment using the 24-h recall questionnaire due to the misreporting of food intake and the fact of not being too precise, what can cause bias to the results. In future surveys, it would be important to collect blood pressure data, given its possible relation with Na^+^ and K^+^ intake.

## Conclusion

In conclusion, this study reports a lower K^+^ intake estimated by 24-h urine excretion than the recommended for 8–10 year-old children. This data shows the need towards health promotion interventions to broaden public awareness in order to increase K^+^ intake.

## Abbreviations

AI: Adequate intake
BMI: Body mass index
BMR: Basal metabolic rate
BP: Blood pressure
DRI: Dietary reference intakes
EI: Energy intake
FCT: Fundação para a Ciência e Tecnologia
NCD: Noncommunicable disease
PAL: Physical activity level
WHO: World Health Organization
