# Erratum to: Potassium urinary excretion and dietary intake: a cross-sectional analysis in 8–10 year-old children

**DOI:** 10.1186/s12887-016-0562-5

**Published:** 2016-02-11

**Authors:** Ana Catarina Oliveira, Patrícia Padrão, André Moreira, Mariana Pinto, Mafalda Neto, Tânia Santos, Joana Madureira, Eduardo de Oliveira Fernandes, Pedro Graça, João Breda, Pedro Moreira

**Affiliations:** Faculty of Nutrition and Food Sciences, University of Porto, R. Dr. Roberto Frias, Porto, 4200-465 Portugal; Institute of Public Health – University of Porto (ISPUP), Porto, Portugal; Department of Immunology, Faculty of Medicine, University of Porto, Porto, Portugal; Department of Immunoallergology, Hospital of São João, Rua Prof. Hernâni Monteiro, Porto, 4200-319 Portugal; Faculty of Sciences, University of Porto, Porto, Portugal; Institute of Mechanical Engineering, Faculty of Engineering, University of Porto, Porto, Portugal; Directorate General for Health (Direcção Geral de Saúde), Lisbon, Portugal; Division of Noncommunicable Diseases and Life-course, WHO Regional Office for Europe, UN City, Copenhagen, Denmark; Research Centre on Physical Activity and Health, University of Porto, Rua Dr. Plácido Costa, 91, Porto, 4200-450 Portugal

After publication of the original article [1] we were contacted by the authors asking that the funding information be updated. The updated text should read as:

This work was supported by ARIA Project PTDC/DTP-SAP/1522/2012 from Foundation for Science and Technology (Fundação para a Ciência e Tecnologia - FCT) co-financed by European Regional Development Fundthrough Operational Competitiveness Programme (COMPETE) FCOMP −01-0124-FEDER-028797; and by the Portuguese CCDR-N for funding the research project “E2BE” (NORTE-07-0124- FEDER-000036), through the European Union FEDER programme.
